# Genomic characterization of multidrug-resistance gene *cfr* in *Escherichia coli* recovered from food animals in Eastern China

**DOI:** 10.3389/fmicb.2022.999778

**Published:** 2022-09-08

**Authors:** Biao Tang, Juan Ni, Jiahui Lin, Yangying Sun, Hui Lin, Yuehong Wu, Hua Yang, Min Yue

**Affiliations:** ^1^State Key Laboratory for Managing Biotic and Chemical Threats to the Quality and Safety of Agro-Products, Institute of Agro-product Safety and Nutrition, Academy of Agricultural Sciences, Hangzhou, China; ^2^School of Food and Pharmacy, Ningbo University, Ningbo, China; ^3^College of Life Sciences and Medicine, Zhejiang Sci-Tech University, Hangzhou, China; ^4^Department of Veterinary Medicine, Institute of Preventive Veterinary Sciences, Zhejiang University College of Animal Sciences, Hangzhou, China; ^5^Hainan Institute of Zhejiang University, Sanya, China

**Keywords:** *Escherichia coli*, florfenicol, cfr, antimicrobial resistance, circular intermediate

## Abstract

The plasmid-borne *cfr* gene, mediating multiple drug resistance (MDR), has been observed in many Gram-positive bacteria. The prevalence of *cfr* and its co-occurrence with additional antimicrobial resistance (AMR) determinants in *Escherichia coli* is an ongoing issue. Additionally, the prevalence and transfer mechanism of the *cfr* gene remain partially investigated. Here, eight *cfr*-positive *E*. *coli* strains were screened using PCR from an extensive collection of *E*. *coli* (*n* = 2,165) strains isolated from pigs and chickens in 2021 in China, with a prevalence rate of 0.37%. All of them were MDR and resistant to florfenicol and tetracycline. These strains can transfer the *cfr* gene to *E*. *coli* J53 by conjugation (1.05 × 10^−1^ – 1.01 × 10^−6^). Moreover, the IncX4 plasmid p727A3-62 K-cfr (62,717 bp) harboring *cfr* in strain EC727A3 was confirmed using Oxford Nanopore Technology. The unknown type plasmid p737A1-27K-cfr (27,742 bp) harboring *cfr* in strain EC737A1 was also identified. Notably, it was verified by PCR that three of the eight *E*. *coli* strains were able to form the *cfr*-IS*26* circular intermediate. It was 2,365 bp in length in strains EC727A3 and ECJHZ21-173, and 2,022 bp in length in EC737A1. Collectively, this study demonstrated that IS*26* plays a vital role in transmitting the MDR gene *cfr* in *E*. *coli via* conjugation and provided updated knowledge regarding *cfr* in *E*. *coli* in Eastern China.

## Introduction

Antimicrobial resistance (AMR) is a serious threat to global public health. The capability of bacteria to acquire and transfer antibiotic resistance and virulence genes is dangerous and urgently crucial to both human and animal health. The multidrug-resistance (MDR) gene *cfr* encodes 23S rRNA methylase, which is resistant to five classes of antimicrobials, including phenols, lincosamides, oxazolidinones, pleuromutilin, and streptomycin A class antibiotics (PhLOPSA phenotype) (Kehrenberg et al., 2005; Long et al., 2006), and has decreased susceptibility to the 16-membered macrolides spiramycin, and josamycin (Smith and Mankin, 2008). For the first time, the discovery of multiple AMR gene *cfr* in *Staphylococcus bovis* isolates has attracted attention in a global sense (Schwarz et al., 2000). Insertion sequences and transposons are associated with the spread of *cfr* in Gram-negative and Gram-positive bacteria, including but not limited to, *Enterococcus*, *Bacillus*, *Jeotgalicoccus*, *Macrococcus*, *Pasteurella multocida*, *Vibrio diabolicus*, *Escherichia coli*, *Streptococcus*, and *Proteus vulgaris* (Dai et al., 2010; Wang et al., 2011, 2012a,b, 2013; Chen et al., 2020a,b; Liu et al., 2022), considering that *cfr* is usually located on plasmids containing related insertion sequences and transposons (Shen et al., 2013; Partridge et al., 2018).

Based on published articles to date, a total of 112 strains of *E*. *coli* containing the MDR gene *cfr* have been identified in various provinces of China; the primary source of these *E*. *coli* strains are pigs, which may be related to the overuse of florfenicol for disease prevention and treatment in pig farms (Wang et al., 2012a, 2018; Zhang et al., 2014, 2015, 2016; Liu et al., 2017; Ma et al., 2021; Tang et al., 2021). For example, it coexists with the extended-spectrum-β-lactamase gene *bla*_CTX-M-14b_, tigecycline resistance gene *tet*(X4), colistin resistance gene *mcr-1*, and florfenicol resistance gene *floR* (Zhang et al., 2015; Ma et al., 2021; Tang et al., 2021). These plasmids carrying the *cfr* gene in *E*. *coli* belong to the plasmid replicon type, including IncX4, IncA/C, IncF14: A-: B-, IncN-IncX1 (Zhang et al., 2014; Sun et al., 2015; Wang et al., 2018; Tang et al., 2021), of which, IncX4 plasmids are frequently detected in China (Wang et al., 2018). However, few studies have investigated the mechanisms of transmission of the MDR gene *cfr* in *E*. *coli*.

In this study, the prevalence and characteristics of *E*. *coli cfr*-positive strains in food animals were investigated. All *cfr*-positive strains were further sequenced by Illumina or Nanopore platforms, and the *cfr*-harboring plasmids were also identified and characterized. It was confirmed that circular intermediate and conjugation transfer promoted the transfer of the *cfr* gene. Our study highlights the severe threat posed by *cfr*-carrying *E*. *coli* to public health and provides new insight on its role in dissemination.

## Materials and methods

### Screening of the *cfr* gene

From May to December 2021, 2,103 *E*. *coli* strains were isolated from 11 cities in Zhejiang, including Hangzhou, Jinhua, Jiaxing, Qvzhou, Ningbo, Taizhou, Shaoxing, Zhoushan, Lishui, Wenzhou and Huzhou, including 1,186 strains from pigs, 904 from strains in chickens and 13 strains from ducks. Thirty-six *E*. *coli* strains were isolated from Jiangxi Province, 25 *E*. *coli* strains were isolated from Hunan Province, and one was isolated from Anhui Province (Table 1). PCR screening of isolated strains was performed to obtain the prevalence of the *cfr* gene in the above *E*. *coli* isolates with primer sequences (F: GTGAAGCTCTAGCCAACCGTC; R: GCAGCGTCAATATCAATCCC), as described previously (Osman et al., 2019).

**Table 1:** Strain information for screening the *cfr* gene.

Province	Animal	Number
Zhejiang	Pig	1,186
	Chicken	904
	Duck	13
Jiangxi	Duck	36
Hunan	Chicken	25
Anhui	Pig	1
Total	–	2,165

### Antimicrobial Susceptibility Test

*Escherichia coli* was inoculated on Luria-Bertani (LB) agar medium for pure culture, according to the micro-dilution method recommended in the M100-S31 document of the American Committee for Clinical Laboratory Standardization (CLSI) (Humphries et al., 2021; Tang et al., 2022b). The antimicrobial susceptibility of *E*. *coli* to 13 tested antibiotics were, ampicillin (2–128 μg/ml), amoxicillin-clavulanate acid (4/2–128/64 μg/ml), cefotaxime (0.06–64 μg/ml), meropenem (0.5–16 μg/ml), amikacin (2–64 μg/ml), gentamicin (0.25–32 μg/ml), colistin (0.125–8 μg/ml), ceftiofur (0.25–32 μg/ml), ciprofloxacin (0.06–8 μg/ml), trimethoprim-sulfamethoxazole (0.5/9.5–16/304 μg/ml), tetracycline (0.25–64 μg/ml), tigecycline (0.25–32 μg/ml), and florfenicol (2–128 μg/ml). *E*. *coli* ATCC 25922 served as quality control bacteria.

### Whole-genome sequencing

To further understand the genetic background of themultiple AMR gene *cfr* in *E*. *coli*, a genomic DNA extraction kit (Generay, Shanghai, China) was used to extract bacterial genomic DNA from all *cfr* positive strains for whole-genome sequencing (WGS). An Illumina sequencing library was generated using the NEXTflex DNA sequencing kit (Bioo Scientific, Austin, United States). Illumina paired-end sequencing was performed using the HiSeq-PE150 strategy, and the readings were filtered using fastp v0.12. Clean data were reconstructed using CLC Genomic Workbench 12.0. Prototypical strains were simultaneously whole-genome sequenced on the Oxford Nanopore GridION platform (Oxford, United Kingdom). The above genomic DNA library was prepared using the SQKLSK109 kit (Oxford Nanopore Technologies, Oxford, United Kingdom). Guppy v3.2.4 was used for base invocation and removal of adapter sequences. Sequences were assembled from scratch using a mixture of short and long reads from the Unicycler v0.4.4 pipeline (Wick et al., 2017). The reconstruction of plasmids from next generation sequence pair-end datasets was performed by PLACNETw (Vielva et al., 2017).

### Antimicrobial resistance gene, virulence gene, phylogenetic tree and plasmid analysis

Acquired AMR genes and chromosomal mutations were predicted using ResFinder 4.1^1^ with a percentage identification threshold of 90% and a minimum coverage length of 60%. The virulence genes were predicted using VirulenceFinder 2.0.^2^ Plasmid replicon type identification using PlasmidFinder 2.1^3^ with a percentage identification threshold of 95% and percentage coverage length of 60%. Multilocus sequence typing (MLST) was performed using MLST 2.0.^4^ Phylogenetic analysis of genomes and plasmids based on maximum likelihood was performed using kSNP3 (Gardner et al., 2015). Easyfig 2.2.3 was used to compare the gene–environment (Sullivan et al., 2011). BRIG was used to plot circles of multiple plasmids for comparison (Alikhan et al., 2011).

### Conjugation transfer assay

The *E*. *coli* strain J53 was selected as the recipient strain, and *cfr*-positive *E*. *coli* was selected as the donor strain. Florfenicol and sodium azide were added for the selection. First, we determined that *cfr*-positive *E*. *coli* could not be grown on LB plates containing 100 mg/l sodium azide, and J53 could no longer be grown on LB plates containing10 mg/l florfenicol. The method of conjugation transfer was mentioned in previous reports (Xu et al., 2021; Tang et al., 2022a). The donor bacteria and recipient bacteria were inoculated into LB broth and cultured on a shaker for 4–6 h. One milliliter of the bacterial solution was taken for centrifugation, and the donor and recipient bacteria were added to the LB plate overnight at 37°C. After gradient dilution with PBS, they were inoculated onto LB square plates containing 10 mg/l florfenicol and 100 mg/l sodium azide. Finally, single colonies that grew after mating were identified *via* PCR to exclude false-positive cases.

### Detection of IS*26*-mediated circularization with a *cfr*-containing gene

To verify the circularization potential of the IS*26* flanking fragments in a plasmid, a pair of primers were designed and amplified by PCR to observe whether they could form the circular intermediate of *cfr*-IS*26*. The primers used to identify the *cfr*-IS26 circular intermediate are shown (F: GTTGCCTGGTGTAAATGATTC; R: CTGCTAAGAGCTTGATATTC). The size of the *cfr*-IS*26* circular intermediate was determined by Sanger sequencing.

## Results

### Antimicrobial susceptibility test of *E*. *coli* carrying the *cfr* gene

**Figure 1:**
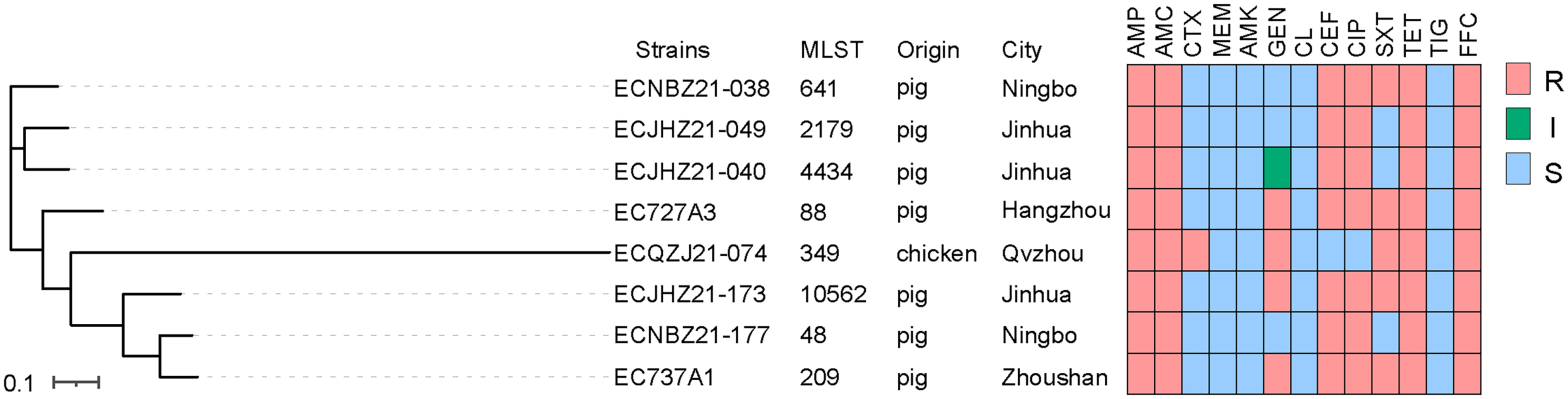
The phylogenetic tree and AMR of *E*. *coli* isolates. ‘S’ represents susceptibility, ‘I’ represents intermediate, and ‘R’ represents resistance. Penicillins: AMP, ampicillin; AMC, amoxicillin-clavulanic acid; Cephalosporins: CTX, cefotaxime; CEF, ceftiofur; Carbapenems: MEM, meropenem; Aminoglycosides: AMK, amikacin; GEN, gentamicin; Polypeptides: CL, colistin; Quinolones: CIP, ciprofloxacin; Sulfonamides: SXT, trimethoprim-sulfamethoxazole; Tetracyclines: TET, tetracycline; TIG, tigecycline; Chloramphenicol: FFC, florfenicol.

Eight *cfr*-positive isolates were identified from 2,165 *E*. *coli* isolates (1,187 from pigs, 929 from chickens, and 49 from ducks), and the prevalence was 0.37% (Table 2). Seven of the *cfr*-positive strains were isolated from pigs, and one strain was isolated from chicken. The AST results of eight positive *E*. *coli* isolates showed that all strains were resistant to ampicillin, amoxicillin-clavulanic acid, tetracycline, and florfenicol (Figure 1; Supplementary Table 1). All the strains were sensitive to colistin, meropenem, tigecycline, and amikacin.

**Table 2:** *cfr*-positive *E*. *coli* isolates in this study.

Strains	Source	Animal	City	Plasmid	Accession number
ECJHZ21-040	Feces	Pig	Jinhua	–	JAMYDT000000000
ECJHZ21-049	Feces	Pig	Jinhua	–	JAMYDS000000000
ECNBZ21-038	Feces	Pig	Ningbo	–	JAMYDR000000000
ECNBZ21-177	Feces	Pig	Ningbo	–	JAMYDQ000000000
ECJHZ21-173	Feces	Pig	Jinhua	–	JAMYDP000000000
ECQZJ21-074	Feces	Chicken	Qvzhou	–	JAMYDO000000000
EC727A3	Feces	Pig	Hangzhou	p727A3-62K-cfr	CP100062-CP100071
EC737A1	Gut	Pig	Zhoushan	p737A1-27K-cfr	CP100005-CP100012
ECJHZ21-058	Feces	Pig	Jinhua	–	JAMYDT000000001

### Molecular characterization and conjugative transfer of *cfr*-positive isolates

The contigs carrying *cfr* gene assembled by the second generation sequence are between 1 and 3 Kb in length (Supplementary Figure 1). The *cfr*-harboring *E*. *coli* strains isolated from chicken and pig belonged to different branches. Among the strains ECJHZ21-040, ECJHZ21-049, and ECNBZ21-038 were clustered together. Additionally, ECQZJ21-074 belonged to independent lineages, and there were differences between them and in the seven strains mentioned above (Figure 1). The eight *E*. *coli* isolates had distinct sequence types (STs) with ST641, ST2179, ST4434, ST88, ST349, ST10562, ST48, and ST209, indicating that *cfr* was widely distributed in *E*. *coli* with different genetic backgrounds.

**Figure 2:**
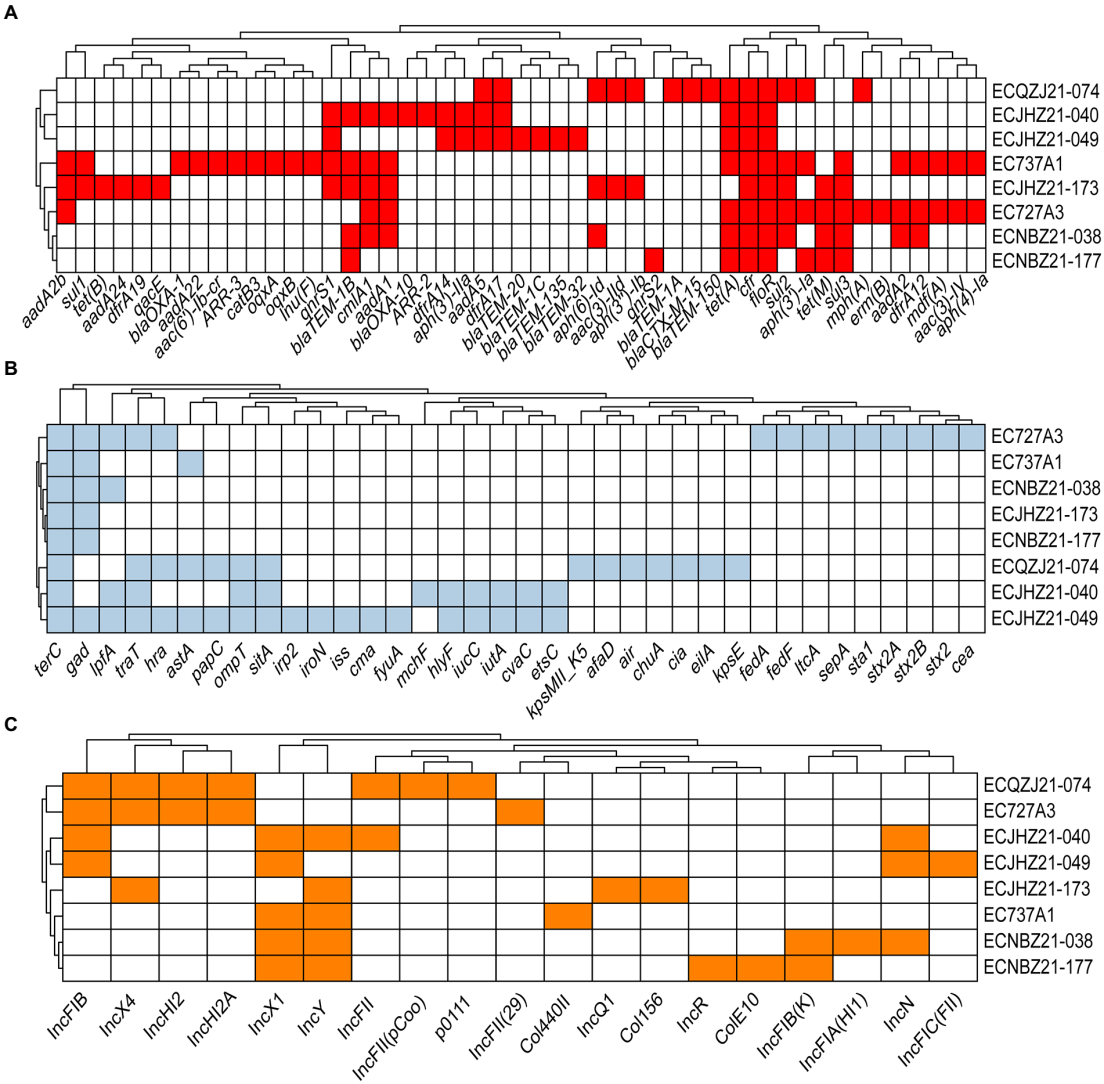
The AMR genes, plasmid replicons, and virulence genes in *cfr*-positive *E*. *coli*. **(A)** Acquired AMR gene in *cfr*-positive *E*. *coli*. Red indicates the AMR gene. **(B)** Virulence gene in *cfr*-positive *E*. *coli*. Light blue represents the presence of the virulence gene, and white represents the absence of the virulence gene. *terC*, tellurium ion resistance; *gad*, glutamate decarboxylase; *lpfA*, long polar fimbriae; *traT*, outer membrane protein complement resistance; *hra*, heat-resistant agglutinin; *astA*, EAST-1 heat-stable toxin; *papC*, outer membrane usher P fimbriae; *ompT*, outer membrane protease; *sitA*, iron transport; *irp2*, high molecular weight protein 2 non-ribosomal peptide synthetase; *iroN*, enterobactin siderophore receptor; *iss*, increased serum survival; *cma*, colicin M; *fyuA*, siderophore receptor; *mchF*, ABC transporter; hlyF, hemolysin F; *iucC*, aerobactin synthetase; *iutA*, ferric aerobactin receptor; *cvaC*, microcin C; *etsC*, putative type I secretion outer membrane; *kpsMII_K5*, polysialic acid transport; *afaD*, afimbrial adhesion; *air*, enteroaggregative immunoglobulin; *chuA*, outer membrane hemin receptor; *cia*, colicin ia; *eilA*, salmonella HilA homolog; *kpsE*, capsule polysaccharide export inner-membrane; *fedA*, fimbrial protein F107 subunit A; *fedF*, fimbrial adhesin AC precursor; *ltcA*, heat-labile enterotoxin A subunit; *sepA*, shigella extracellular protein A; *sta1*, Heat-stabile enterotoxin ST-Ia; *stx2A*, shiga toxin 2, subunit A; *stx2B*, shiga toxin 2, subunit B; *stx2*, O139 S1191, variant e; *cea*, colicin E1. **(C)** Plasmid replicon type in *cfr*-positive *E*. *coli*. Orange represents the plasmid replicon type; white represents none of the genes predicted.

A total of 49 types of AMR determinants within 10 classes of antibiotics were detected (Figure 2A). In addition, there were two florfenicol genes (*cfr*, *floR*), three tetracycline genes (*tet*(A), *tet*(B), and *tet*(M)), 10 β-lactam genes (*bla*_CTX-M-15_, *bla*_TEM-150_, *bla*_TEM-1A_, *bla*_TEM-1B_, *bla*_OXA-10_, *bla*_TEM-1C_, *bla*_OXA-20_, *bla*_OXA-135_, *bla*_TEM-32_, *bla*_OXA-1_), two quinolone genes (*qnr*S1, *qnr*S2), two rifamycin genes (*ARR*-2, *ARR*-3), three macrolide genes (*mph*(A), *mdf*(A), and *erm*(B)), one lincosamide gene (*Inu*(F)), six folate pathway antagonist genes (*sul*1, *sul*2, *sul*3, *dfrA*12, *dfrA*17, *dfrA*19), 14 aminoglycoside genes (*aad*A2b, *aph(4)-Ia*, *aac(3)-IV*, *aadA*2, *aph(3′)-Ia*, *aph(3″)-Ib*, *aac(3)-IId*, *aph(6)-Id*, *aad*A5, *aph(3′)-IIa*, *aad*A1, *aac(6′)-Ib-cr*, *aad*A22, *aad*A24) and some additional AMR determinants (Figure 2A). The virulence genes of the strains included *terC*, *traT*, *gad*, *lpfA*, *ompT*, *sitA*, *astA*, *hra*, etc. (Figure 2B). Among them, *astA* is a virulence gene encoding heat-stable enterotoxin of enteroaggregative *E*. *coli*, which may produce related toxins with the possibility of pathogenicity. Importantly, strain EC727A3 contains the virulence genes *stx*2A and *stx*2B that produce Shiga toxin, which may cause self-limiting diarrhoeal disease and sometimes bloody diarrhea as well as complications such as hemorrhagic colitis and hemolytic uremic syndrome (HUS) (Fitzpatrick, 1999; Launders et al., 2016; Mcfarland et al., 2017). Plasmid replicons include 19 types such as IncFIC(FII), IncN, IncFIA(HI1), IncFIB(K), ColE10, IncR, Col156, IncQ1, Col440II, IncFII(29), p0111, IncFII(pCoo), IncFII, IncY, IncX1, IncHI2A, IncHI2, IncX4, and IncFIB. The plasmid types of the eight isolates remained genetically diverse (Figure 2C).

The conjugation transfer assay demonstrated that all transconjugants from *cfr*-positive *E*. *coli* strains and *E*. *coli* J53 could grow normally on LB plates containing 100 mg/l sodium azide and 10 mg/l florfenicol. Further, PCR confirmed that the transconjugant contained the *cfr* gene, which indicated that the conjugative transfer experiment was successful, with a transfer frequency of 1.05 × 10^−1^–1.01 × 10^−6^.

### Genetic environment of the *cfr*-positive isolates.

**Figure 3:**
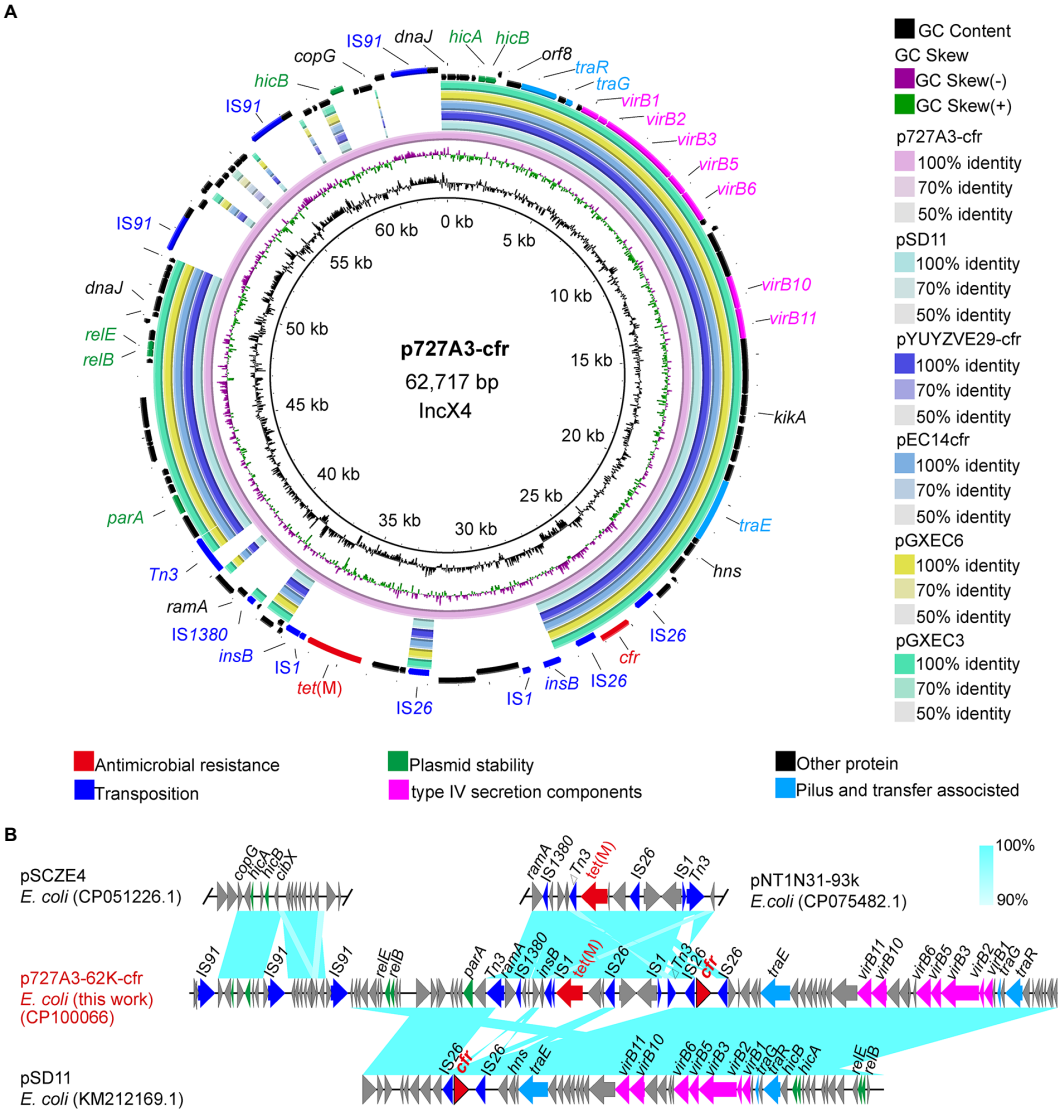
Sequence alignment of plasmid p727A3-62K-cfr and the gene–environment of *cfr*. **(A)** Comparison of the plasmid sequences between p727A3-62K-cfr, pSD11, pYUYZVE29-cfr, pEC14cfr, pGXEC6, and pGXEC3 of *E*. *coli* strains. **(B)** Linear comparison of the plasmid sequences of *E*. *coli* p727A3-62K-cfr and *E*. *coli* pSCZE4, pSD11, pNT1N21-93k. Open arrows indicate coding sequences (red arrows, AMR genes; green arrows, plasmid replication; blue arrows, transfer and transfer-related sequences; gray arrows, hypothetical, and unclassified) and indicate the direction of transcription.

Two isolates were randomly selected from the eight *cfr*-positive strains for nanopore sequencing to obtain their complete genome sequences. To understand how *cfr* is transmitted, the genetic background of the *cfr* gene was further investigated. The *cfr* gene was located on the IncX4-type plasmid p727A3-62K-cfr (CP100066) in strain EC727A3. The length of p727A3-62K-cfr was 62,717 bp, and the GC content was 44% (Figure 3A). Moreover, p727A3-62K-cfr had high similarity with another *cfr*-carrying plasmid and had the highest homology with plasmid pSD11 (KM212169.1, 37,672 bp) from porcine *E*. *coli* strain 8ZG6D (65% query coverage and 99.99% identity). The collinear comparison showed that p727A3-62K-cfr and pSD11 had two different gene arrangements. The 12,647 bp region had high homology with the sequence containing the *tet*(M) gene in pNT1N31-93k (CP075482, 93,332 bp), and there was an insertion sequence IS*1* upstream of *tet*(M) compared with pNT1N31-93k (Figure 3B). The other 10,831 bp region had a higher homology to a part of pSCZE4 (CP051226, 60,732 bp), and this sequence had three more IS*91* insertion sequences in the same direction than pSCZE4 (Figure 3B).

**Figure 4:**
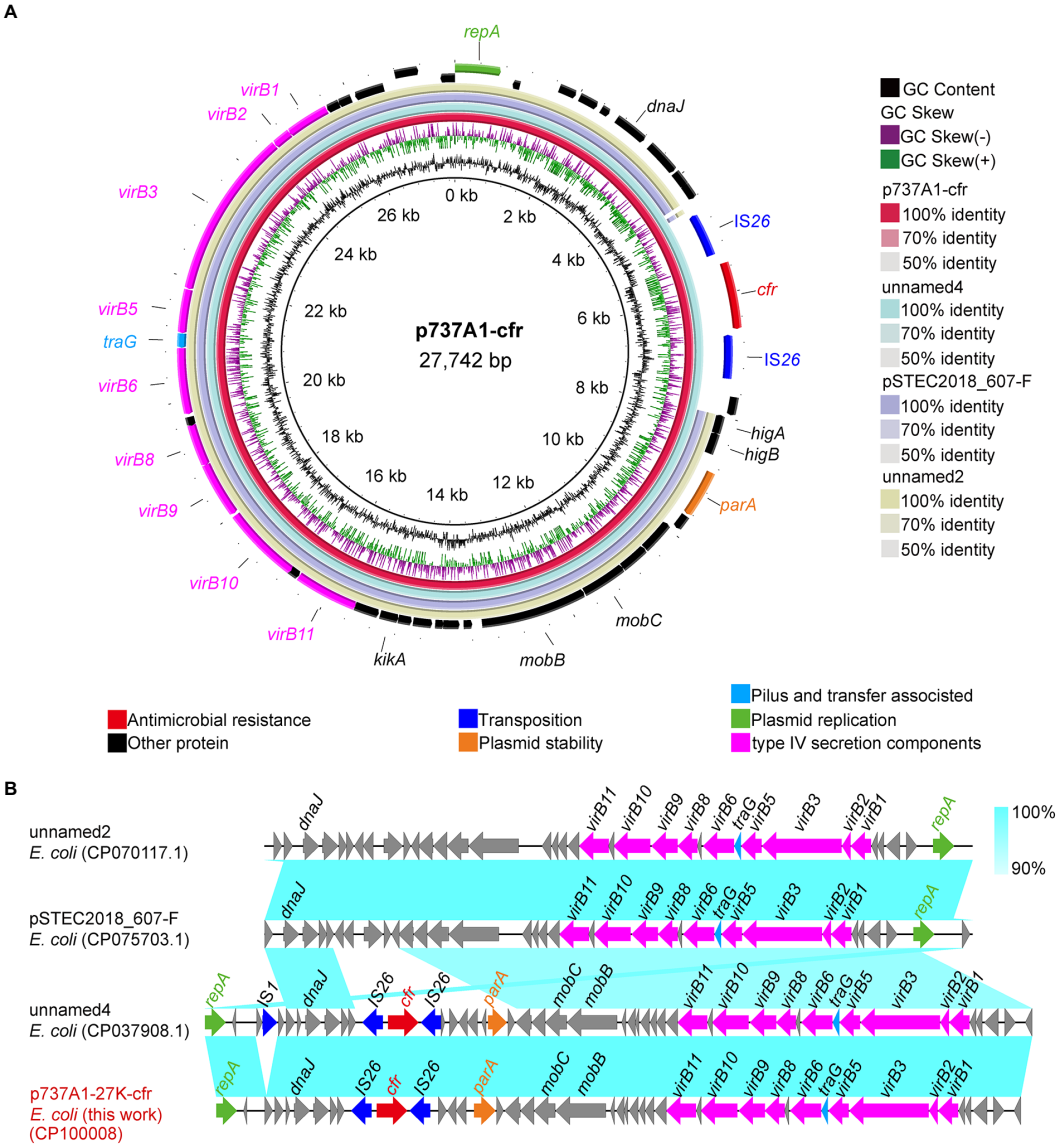
Sequence alignment of plasmid p737A1-62K-cfr and the gene–environment of *cfr*. **(A)** Comparison of the plasmid sequences between *E*. *coli* p737A1-27K-cfr, *E*. *coli* plasmid unnamed4, pSTEC2018_607-F, unnamed2, FHI99-scaffold-18_conting-14, and pEC2547-KPC-2. **(B)** Linear comparison of the plasmid sequences of *E*. *coli* p737A1-27K-cfr and *E*. *coli* plasmid unnamed4, pSTEC2018_607-F, and plasmid unnamed2. Open arrows indicate coding sequences (red arrows, AMR genes; green arrows, plasmid replication; blue arrows, transfer, and transfer-related sequences; gray arrows, hypothetical, and unclassified) and indicate the direction of transcription.

The *cfr* gene of strain EC737A1 was located on plasmid p737A1-27K-cfr (CP100008). The length of p737A1-27K-cfr was 27,742 bp, and the GC content was 43% (Figure 4A). Plasmid p737A1-27K-cfr had a high degree of homology (100% query coverage and 100% recognition) with plasmid unnamed4 (CP037908.1, 28,519 bp). The collinear comparison showed that a 777 bp region containing the IS*1* mobile element was inserted into the plasmid p737A1-27K-cfr to form unnamed4. However, the type of plasmid had not yet been determined; it was only known that the backbone of plasmid p737A1-27K-cfr was derived from pSTEC2018_607-F (CP075703.1, 24,412 bp). The 4,270 bp construct containing the IS*26*-*cfr*-IS*26*-*higA*-*higB*-*parK* was inserted into the plasmid pSTEC2018_607-F (Figure 4B).

### *cfr*-IS*26* circular intermediate

**Figure 5:**
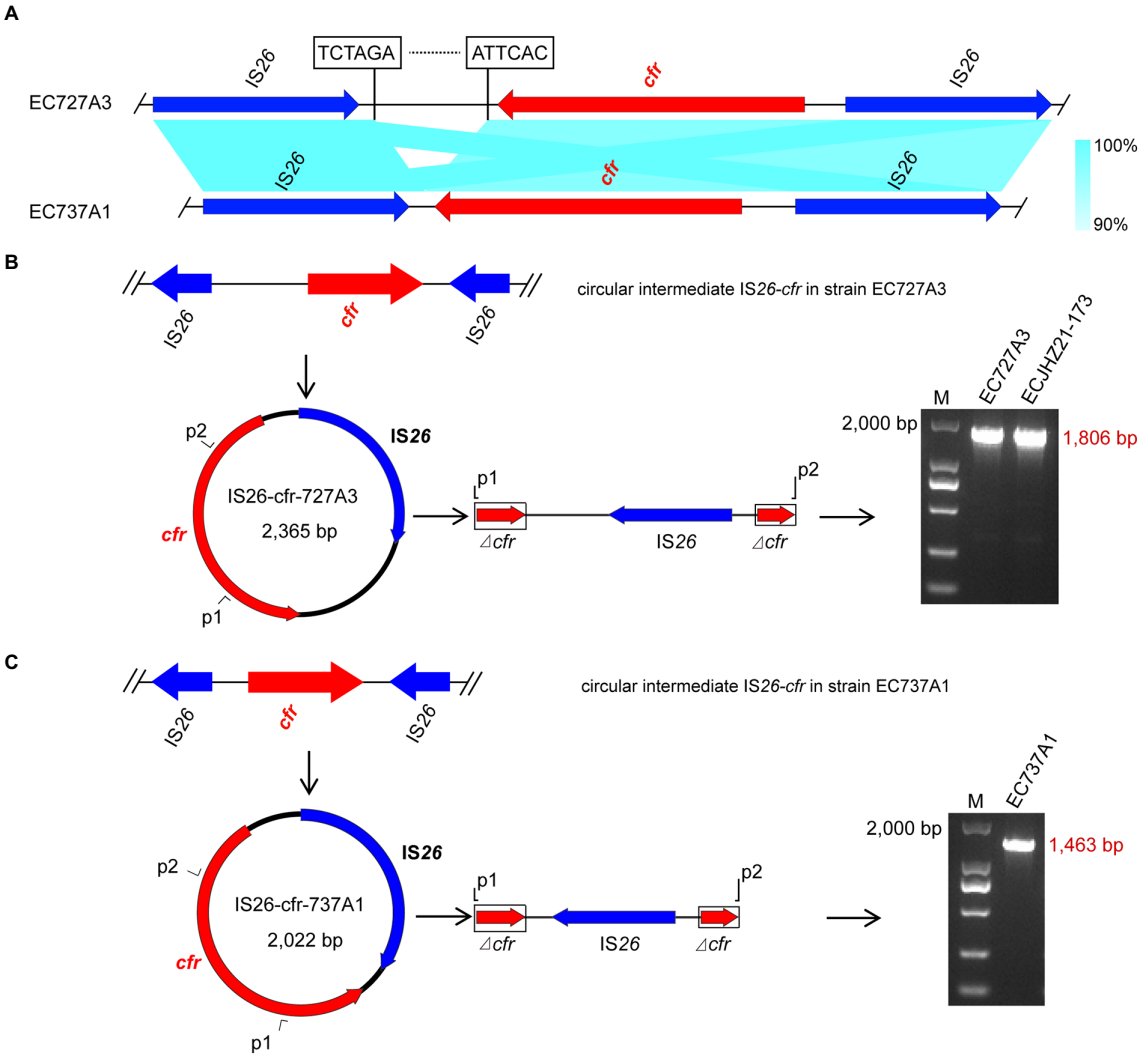
Structures of IS*26*-*cfr* circular intermediates in EC727A3 and EC737A2. **(A)** Linear comparison of IS*26*-*cfr*-IS*26* genomic sequences in *cfr*-positive *E*. *coli* EC727A3 and EC737A1. **(B)** Circular intermediates formed by the *cfr* gene in EC727A3 and the size of amplicons of *cfr* circular intermediates obtained by gel electrophoresis. **(C)** Circular intermediates formed by the *cfr* gene in EC737A1 and the size of amplicons of *cfr* circular intermediates obtained by gel electrophoresis.

Genome analysis found that both the upstream and downstream regions of the *cfr* gene in EC727A3 and EC737A1 had an IS*26* element in the same direction, forming an IS*26*-*cfr*-IS26 structure (Figure 5A). However, there was a 343 bp size difference between the IS26-*cfr*-IS26 structures in EC727A3 and EC737A1. PCR determined that three out of eight *E*. *coli* strains could form *cfr*-IS*26* cyclic intermediates of two different sizes. Among them, the size of the circular intermediate formed by ECJHZ21-173 and EC727A3 was the same, at 2,365 bp (Figure 5B). The size of the *cfr*-IS*26* circular intermediate in EC737A1 was 2,022 bp (Figure 5C).

## Discussion

To date, the prevalence of the *cfr* gene in *E*. *coli* from animals has been reported to be 0.37% in Eastern China. In previous studies, most of the *cfr* genes in *E*. *coli* were isolated from pigs (Deng et al., 2014; Zhang et al., 2014). As far as we know, only four *E*. *coli* strains of chicken origin containing the *cfr* gene have been identified in Guangdong Province, Fujian Province and Heilongjiang Province (Zhao et al., 2016; Wang et al., 2018). No *cfr* gene has been found in human clinical *E*. *coli* isolates. In this study, we isolated the *cfr* gene from chicken sources in addition to pigs, and the prevalence of the *cfr* gene in *E*. *coli* isolates was higher than the initially reported at 0.08% (1/1230) (Wang et al., 2012a). This was similar to the previously reported 0.5% (2/398) (Liu et al., 2017) but much lower than the 13.7% (85/617) recently reported in Guangdong Province, China (Ma et al., 2021). According to the official, authoritative statement, in 2018 (P.R., 2019) and 2020 (P.R., 2021), the use of phenicols was 2,123 and 3,519 tons in animal breeding in China, respectively, and florfenicol was the primary antimicrobials in phenicols used in livestock and poultry breeding (Van Cuong et al., 2016). Previous global or national reports show that the florfenicol resistance gene is related to the long-term use of florfenicol (Li et al., 2020). Our study indicated that the *cfr* gene dissemination was significantly different in different provinces of China, and there was a possibility of rapid spread in a small area.

IS*26* is a universal mobile element in various gram-negative bacteria, including *E*. *coli*, *P. multocida*, *Acinetobacter baumannii*, *Klebsiella pneumoniae*, *V. diabolicus*, and *Proteus vulgaris* (Post and Hall, 2009; Harmer et al., 2014; Chen et al., 2020a; Jin et al., 2021; Zhao et al., 2021). The presence of transfer elements plays a vital role in the transfer of the *cfr* gene. Previous studies confirmed the existence of different genetic environments for the *cfr* gene in *E*. *coli*, with one IS*26* element on each side of the *cfr* gene being the most reported genetic environment in *E*. *coli* and the other two being one IS*256* element on each side of *cfr* and one IS*15* element on each side (Wang et al., 2012a, 2018; Zhang et al., 2016; Liu et al., 2017; Tang et al., 2021). In addition, IS*26* was found to form circular intermediates mediating the transmission of *cfr* genes in *V. diabolicus*. Similarly, it was also found to form circular intermediates that mediate the transmission of other AMR genes in *E*. *coli* (Zhao et al., 2021; Liu et al., 2022). The current study results were inconsistent with previous studies verifying that *cfr* can form a circular intermediate of IS*26*-*cfr* during transmission and facilitate its transmission in *E*. *coli*.

Plasmid p727A3-62K-cfr obtained in the present study belonged to the IncX4 type. The IncX4 plasmids carrying the *cfr* gene have been found in *E*. *coli* isolated from Jiangsu, Guangdong, Guangxi, Liaoning, Jilin, and Heilongjiang Provinces in China (Deng et al., 2014; Mei et al., 2021). This result indicated that the IncX4-type plasmid might be a common plasmid carrying the MDR gene *cfr*. In addition, we also identified a plasmid p737A1-27K-cfr that had not yet been typed, which indicates that the types of plasmids carrying the *cfr* gene are gradually increasing, and it is necessary to pay close attention to the spread of the *cfr* gene in *E*. *coli*.

## Conclusion

Eight strains containing the *cfr* gene were isolated from 2,165 strains of *E*. *coli* in 2021, seven strains were isolated from pig farms, and one strain was isolated from chicken farms, indicating that the *cfr* gene widely exists in a variety of food animals. An IncX4 type plasmid and an unknown type plasmid were found, and the IS*26*-*cfr*-IS*26* structure was verified to form a *cfr*-IS*26* circular intermediate for propagation. Since the widespread use of antibiotics, particularly florfenicol, may promote the spread of *cfr* genes among animals. It is necessary to strengthen the control of veterinary antibiotics and continuously monitor the spread of the *E*. *coli* multidrug resistance gene *cfr* to reduce the potential public health threat.

## Data availability statement

The names of the repository/repositories and accession number(s) can be found in the article/Table 2.

## Author contributions

BT and MY: conceptualization. HY: funding acquisition. BT, JN, JL, HL, and YW: investigation. JN, JL, and BT: methodology. MY and HY: supervision. JN, HL, and BT: visualization. JN and BT: writing—original draft. All authors have read and agreed to the published version of the manuscript.

## Funding

This work was supported by the Key Research and Development Program of Zhejiang Province (2020C02031), the earmarked fund for China Agriculture Research System (CARS-42-27), the State Key Laboratory for Managing Biotic and Chemical Threats to the Quality and Safety of Agro-products (2010DS700124-ZZ2102), Collaborative Extension Plan of Major Agricultural Technologies in Zhejiang Province (2021XTTGXM03) and Major Special Project for the Construction of Agricultural Product Standardized Production Demonstration Counties (zjny2022001).

## Conflict of interest

The authors declare that the research was conducted in the absence of any commercial or financial relationships that could be construed as a potential conflict of interest.

## Publisher’s note

All claims expressed in this article are solely those of the authors and do not necessarily represent those of their affiliated organizations, or those of the publisher, the editors and the reviewers. Any product that may be evaluated in this article, or claim that may be made by its manufacturer, is not guaranteed or endorsed by the publisher.

## Supplementary material

The Supplementary material for this article can be found online at: https://www.frontiersin.org/articles/10.3389/fmicb.2022.999778/full#supplementary-material

## Footnotes

^1^ https://cge.cbs.dtu.dk/services/ResFinder/

^2^ http://cge.cbs.dty.dk/service/VirulenceFinder/

^3^ https://cge.cbs.dtu.dk/services/PlasmidFinder/

^4^ https://cge.cbs.dtu.dk/services/MLST/
