# Supplementary material for: Genomic characterization of multidrug-resistance gene cfr in Escherichia coli recovered from food animals in Eastern China
Source: Front Microbiol. 2022 Sep 8;13:999778. doi: 10.3389/fmicb.2022.999778 (PMC9493366; doi:10.3389/fmicb.2022.999778)
Supplement: Supplementary file 1 [file Table_1.DOCX]

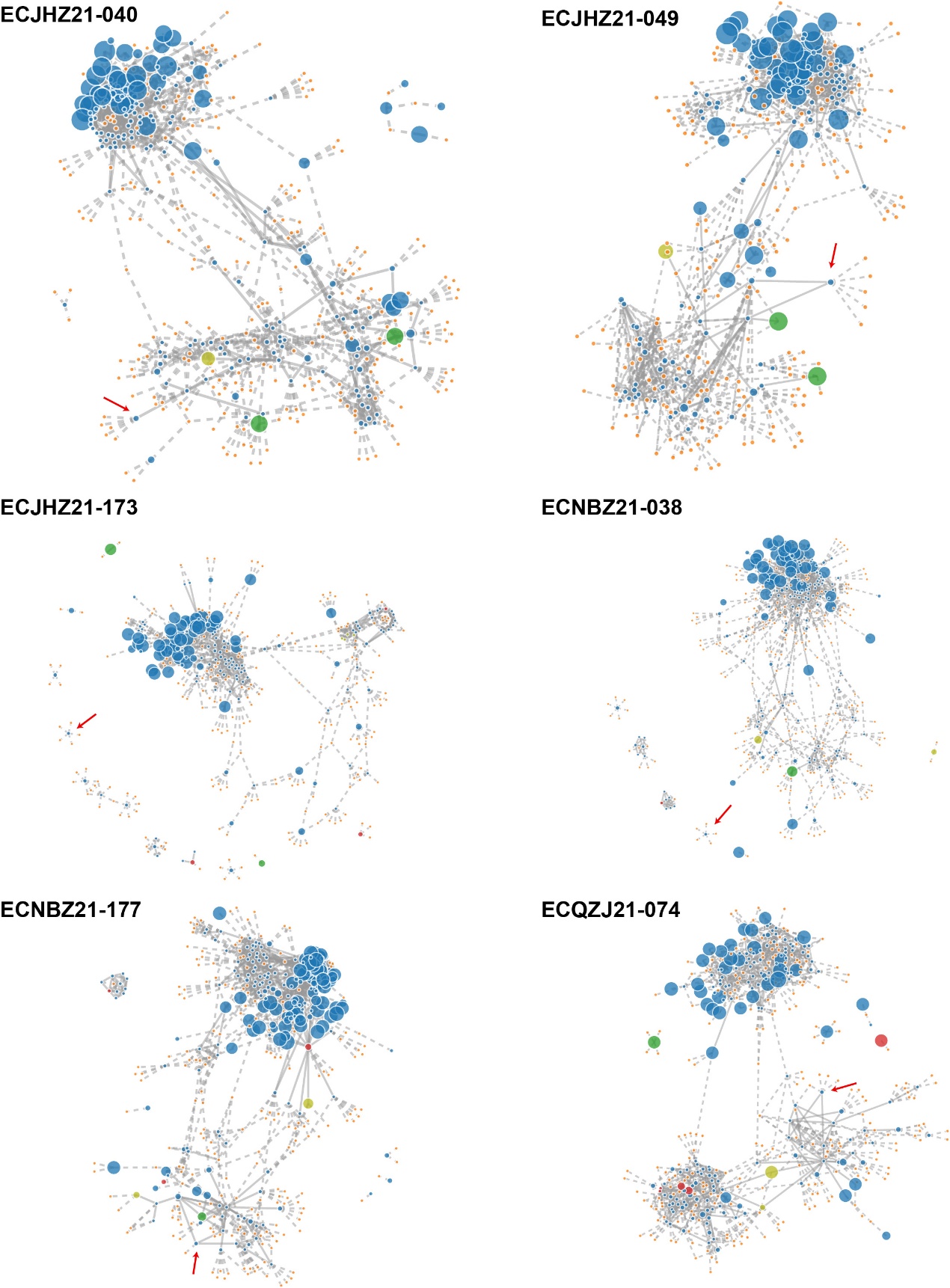


**Figure S1**. The reconstruction of plasmids from next generation sequence pair-end datasets of six *cfr*-harboring *E. coli* strains. The contigs carrying the *cfr* gene are marked with red arrows.

**Table S1.** MIC values of eight *cfr*-positive *E. coli* strains (μg·mL^-1^)

| **Antibiotics** | **ECJHZ21-173** | **ECNBZ21-038** | **ECNBZ21-177** | **ECJHZ21-049** | **ECJHZ21-040** | **ECQZJ21-074** | **EC737A1** | **EC727A3** |
| --- | --- | --- | --- | --- | --- | --- | --- | --- |
| AMP | >128 | >128 | >128 | >128 | >128 | >128 | >128 | >128 |
| AMC | 128/64 | 128/64 | 64/32 | 128/64 | 128/64 | 64/32 | >128/64 | >128/64 |
| CTX | 0.25 | 1 | 0.25 | 0.25 | 0.5 | >8 | 0.25 | 0.5 |
| MEM | 0.5 | 0.5 | 0.5 | 0.5 | 0.5 | 1 | 0.5 | 0.5 |
| AMK | 8 | 4 | 4 | 4 | 4 | 4 | 4 | 2 |
| GEN | >32 | 2 | 2 | 2 | 8 | >32 | 16 | 32 |
| CL | 2 | 1 | 2 | 1 | 1 | 1 | 1 | 2 |
| CEF | 8 | 8 | 8 | >32 | 8 | 1 | >32 | >32 |
| CIP | 4 | 2 | 4 | >8 | 2 | 0.25 | >8 | >8 |
| SXT | >16/304 | >16/304 | 0.5/9.5 | 1/19 | 2/38 | >16/304 | >16/304 | >16/304 |
| TET | >64 | >64 | >64 | >64 | >64 | >64 | >64 | >64 |
| TIG | 2 | 1 | 1 | 1 | 1 | 1 | 1 | 2 |
| FFC | >128 | >128 | >128 | >128 | >128 | >128 | >128 | >128 |
